# β Cell and Autophagy: What Do We Know?

**DOI:** 10.3390/biom13040649

**Published:** 2023-04-04

**Authors:** Hamid-Reza Mohammadi-Motlagh, Mona Sadeghalvad, Niloofar Yavari, Rosita Primavera, Setareh Soltani, Shashank Chetty, Abantika Ganguly, Shobha Regmi, Tina Fløyel, Simranjeet Kaur, Aashiq H. Mirza, Avnesh S. Thakor, Flemming Pociot, Reza Yarani

**Affiliations:** 1 Medical Biology Research Center, Health Technology Institute, Kermanshah University of Medical Sciences, Kermanshah 67155-1616, Iran; 2 Department of Immunology, School of Medicine, Tehran University of Medical Sciences, Tehran 1416634793, Iran; 3 Department of Cellular and Molecular Medicine, The Panum Institute, Faculty of Health and Medical Sciences, University of Copenhagen, 2200 Copenhagen, Denmark; 4 Interventional Regenerative Innovation at Stanford (IRIS), Department of Radiology, Stanford University School of Medicine, Palo Alto, CA 94304, USA; 5 Clinical Research Development Center, Taleghani and Imam Ali Hospital, Kermanshah University of Medical Sciences, Kermanshah 67145-1673, Iran; 6 Translational Type 1 Diabetes Research, Department of Clinical Research, Steno Diabetes Center Copenhagen, 2730 Herlev, Denmark; 7 Department of Pharmacology, Weill Cornell Medicine, New York, NY 10065, USA; 8 Institute for Clinical Medicine, Faculty of Health and Medical Sciences, University of Copenhagen, 2200 Copenhagen, Denmark

**Keywords:** β cell, autophagy, insulin homeostasis, autophagy modulators, type 1 diabetes, type 2 diabetes

## Abstract

Pancreatic β cells are central to glycemic regulation through insulin production. Studies show autophagy as an essential process in β cell function and fate. Autophagy is a catabolic cellular process that regulates cell homeostasis by recycling surplus or damaged cell components. Impaired autophagy results in β cell loss of function and apoptosis and, as a result, diabetes initiation and progress. It has been shown that in response to endoplasmic reticulum stress, inflammation, and high metabolic demands, autophagy affects β cell function, insulin synthesis, and secretion. This review highlights recent evidence regarding how autophagy can affect β cells’ fate in the pathogenesis of diabetes. Furthermore, we discuss the role of important intrinsic and extrinsic autophagy modulators, which can lead to β cell failure.

## 1. Introduction

Diabetes is a disease where no or insufficient insulin is available, or target organs cannot functionally respond to the insulin, which then places “metabolic pressure” on the pancreas. Insulin is produced by β cells, the pancreatic islet’s primary cells, and has a prominent role in glucose homeostasis by regulating blood glucose levels. Failure to control blood glucose results in several complications [1].

Autophagy is vital for maintaining β cell mass and function. However, impaired autophagy seems to harm β cell function through the intracellular accumulation of damaged organelles, such as defective mitochondria, which subsequently causes cell death and reduces β cell mass. It is critical to elucidate the mechanisms of β cell autophagy, its upstream regulators, and the effect of nutrient availability and metabolic hormones in the pathophysiology of diabetes. This review addresses the molecular mechanisms of autophagy in regulating β cell mass and function in diabetes pathogenesis. In addition, we highlight evidence indicating how impaired autophagy contributes to altered crosstalk between β cells and the immune system in the development of diabetes, especially type 1 diabetes (T1D). Finally, the various intrinsic and extrinsic autophagy modulators leading to β cell dysfunction are discussed.

## 2. Autophagy

**Figure 1:**
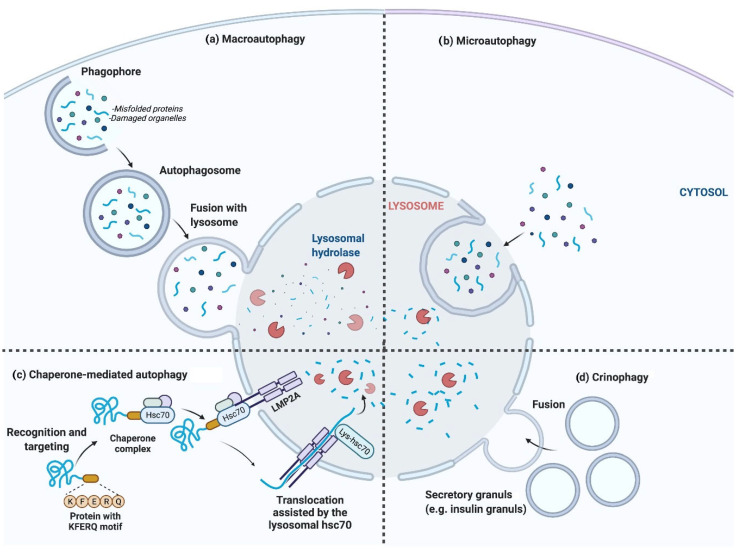
**The** four major types of autophagy. (**a**) Macroautophagy, generally termed autophagy; autophagosomes are formed and engulf cellular components, e.g., secretory granules. Autophagosomes finally fuse with lysosomes, after which the lysosomal hydrolases degrade the sequestered contents. (**b**) Microautophagy, in which cellular components are directly engulfed by the lysosomal membrane. (**c**) Chaperone-mediated autophagy, where specific amino acid sequence motifs of cytosolic proteins are recognized via the chaperone protein hsc70 and directly targeted to lysosomes for degradation. (**d**) Crinophagy, where secretory vesicles directly fuse with lysosomes [6].

Autophagy plays a crucial role in recycling surplus or damaged components of cells, thereby sustaining homeostasis and survival. In autophagy, misfolded proteins and damaged organelles are taken into double-membrane vesicles known as autophagosomes for degradation via lysosomes. This process occurs under the physiological states, including cellular homeostasis, bioenergetic demands, and starvation through protein and organelle turnover. Autophagy can also occur in pathological conditions such as stress, pathogen infection, and other challenging stimuli. In cell starvation, autophagy removes misfolded proteins and deficient organelles, supplying nutrients for ATP synthesis to maintain the cell’s energy level and normal cellular function. However, under specific cellular and environmental conditions, autophagy may also result in cell death through the unregulated degradation of cellular compartments [2,3]. The four major types of autophagy are macroautophagy, microautophagy, chaperone-mediated autophagy, and crinophagy [4,5] (Figure 1).

## 3. Autophagy and β Cell

In β cells, autophagy plays homeostatic roles such as confined apoptosis, preserving insulin secretory granules, and maintaining mitochondrial function [7,8]. β cell mass and function are critical for controlling plasma insulin levels; thus, disturbance in either of these contributes to altered insulin production and secretion failure. Autophagy also contributes to β cell survival during stressful conditions, such as nutrient deprivation, hypoxia, oxidative stress, organelle damage, and endoplasmic reticulum (ER) stress [9,10]. The autophagic response to stress conditions is mainly via the mechanistic target of the rapamycin (mTOR) complex 1 (mTORC1) pathway [10,11,12]. Both inhibition and induction of autophagy through mTORC1 can affect β cell survival and mass [13,14]. Loss of mTORC1, as a critical checkpoint converging cellular stressors, induces impaired autophagy and dysfunction in β cells [13]. The short-term hyperactivation of mTORC1 causes an increase in the mass and function of β cells, including enhanced mitochondrial mass and hyperinsulinemia. Chronic activation of mTORC1 also leads to β cell failure through insulin resistance, autophagy impairment, ER stress, and mitochondrial dysfunction [14].

**Figure 2:**
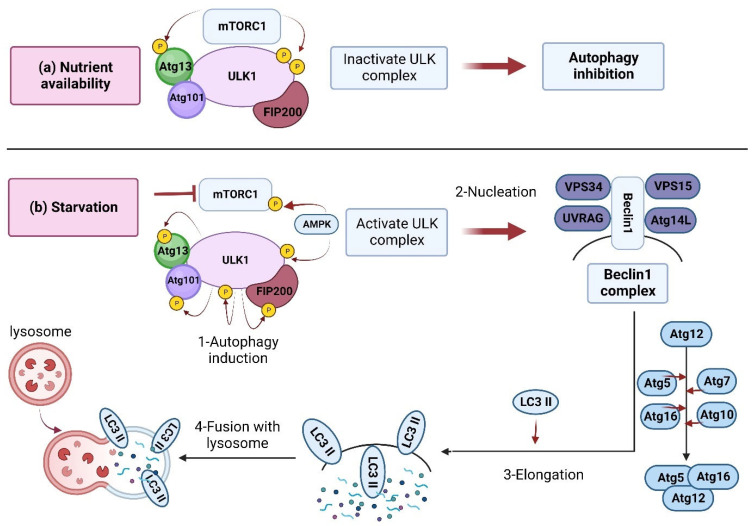
Inhibition and induction of autophagy. (**a**) Nutrient availability: when nutrients are available, mTORC1 is activated and forms a complex accompanied by ULK1, Atg13, Atg101, and FIP200 (200 kDa FAK-family interacting protein) [23]. The developed complex inactivates ULK1 through its phosphorylation, which inhibits autophagy. (**b**) In starvation conditions, mTORC1 is inhibited by dissociation from the ULK1 complex. Meanwhile, the ULK1 is dephosphorylated and activated by AMPK. The activated ULK1 induces autophagy by phosphorylation of Atg13 and FIP200. Then, the Beclin-1 (Bcl-2-interacting protein) is released from Bcl-2 to form a complex with a set of proteins, including Vps34, Vps15, and Atg14L, resulting in autophagosome formation/maturation [23]. This process is accomplished via the Atg proteins in two ubiquitin-like conjugation pathways [24]. Finally, the lipidated LC3 (LC3-II) is localized to the autophagolysosome membrane [25]. LC3 interacts with p62 to engulf ubiquitinated proteins, and within the autophagolysosomes, the lysosomal hydrolases will then degrade the contents.

Under basal conditions (sufficient nutrients), mTORC1 is naturally active and inhibits autophagy. Specific inhibitors, including rapamycin, can induce autophagy by suppressing the mTOR [15,16,17]. In an energy shortage (starvation), several upstream modulators, including AMPK, SIRT1, AKT, and ERK1/2, can regulate autophagy by inhibiting mTORC1 [10,18,19,20,21] (Figure 2). The autophagy-related genes (Atg) have a central role in the autophagy machinery, such as regulating autophagosome formation, a fusion of autophagosomes with lysosomes, and cargo recruitment [22] (Figure 2).

### 3.1. Insulin Homeostasis

The glucose concentration in the blood is the β cells’ initial trigger for insulin secretion [26]. The insulin plasma level is controlled through a clearance mechanism. Autophagy is the main degradation mechanism to remove the excess primary substance of the hormone and prevent overloading at the Golgi during the cell’s resting state. Autophagy is an essential secretory-pathway controller in β cell insulin activity.

Autophagy’s role in insulin biosynthesis and secretion (from proinsulin to insulin) in β cells has been investigated in several studies. Studies on Atg7−/− mice islets [27] showed remarkable amounts of proinsulin aggregated in autophagosomes, where it is degraded via lysosomal activity. Thus, temporary suppression of lysosomal degradation resulted in a significant stable increase in the proinsulin content. Studies using the genetic disruption of autophagy through Atg5/Atg7 knockdown also indicated increased proinsulin content in β cells. Furthermore, electron microscopy studies also demonstrated dispersed, non-granular insulin-like peptides (ILPs) deposited in the autophagosome-like structures. In addition, confocal microscopy of islets isolated from autophagy-deficient β Atg7 knockout mice showed that proinsulin is localized in LC3+ and p62+ spots and protein aggregates, further indicating the regulatory role of autophagy in the proinsulin biosynthesis [27]. In agreement with this, other evidence indicated that autophagy might play a determinant regulatory role in the processing pathway of hormone precursors in the endocrine cells, including β cells. For example, inhibition of autophagy through Atg5/7-knockdown or treatment with the autophagy inhibitor bafilomycin-A1 resulted in significant upregulation of Chromogranin-A (CgA), a precursor of secretory peptide in the INS-1 β cell line [27]. Furthermore, a study showed that treatment with bafilomycin-A1 could also increase proinsulin in the β cells and islets of mice and raise the C-peptide content indicator of proinsulin converting to mature insulin [27].

The Akita mouse is a diabetic animal model. A point mutation changes the A7 cysteine to arginine in proinsulin II, leading to several events such as misfolded proinsulin, severe ER stress, and, finally, β cell failure and cell death, resulting in hyperglycemia. Studies using the diabetic Akita mouse model showed that the proinsulin is deposited in the ER and cannot be accessible to the Golgi and secretory granules, and thus is resistant to lysosomal degradation [28,29,30]. These findings show that the transportation of proinsulin from ER to the secretory pathway may be a prerequisite for its degradation by autophagy compartments. In other words, the drug brefeldin-A, an ER-Golgi transport inhibitor, has been shown to destroy the lysosomal degradation of normal proinsulin.

Further studies showed that the Trans Golgi Network (TGN) protein B4GALT1 is expressed in proinsulin-containing autophagosomes; proinsulin is delivered to autophagosomes at the TGN. However, more studies are needed to determine the exact mechanisms of this molecular event. These findings also show that macroautophagy is the primary degradation mechanism controlling β cell proinsulin. On the other hand, inhibition of autophagy might have a central role in increasing proinsulin biosynthesis in β cells.

**Figure 3:**
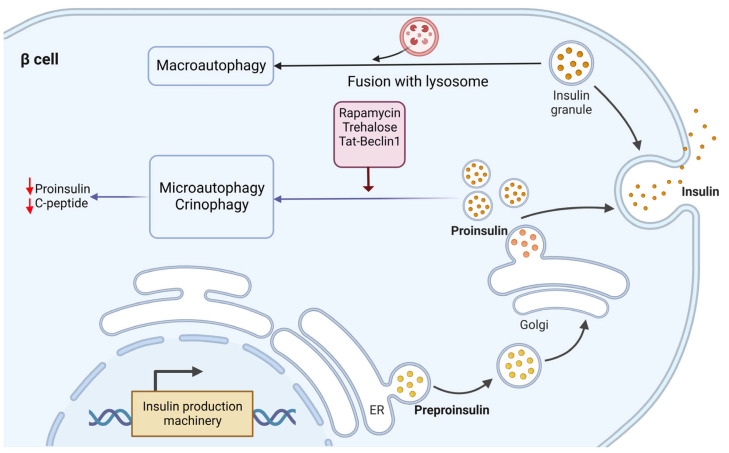
Molecular mechanism of proinsulin and insulin relation with autophagy in ER-Golgi pathway. The degradation mechanism of insulin and proinsulin by lysosomes seem to be different. Proinsulin is mainly degraded via macroautophagy, while insulin granule degradation is through microautophagy and crinophagy. Induction of autophagy by pharmacological stimulators (rapamycin and trehalose) and genetic interference using Tat-beclin-1 exhibited reduced proinsulin content in β cells without affecting the insulin amount.

Investigation of the degradation mechanism behind insulin and its precursor (proinsulin) revealed a difference. While proinsulin is mainly degraded via the macroautophagy mechanism, microautophagy and crinophagy are involved in insulin granule degradation [27] (Figure 3). Recent studies showed that fasting situations guarantee low insulin secretion to avoid hypoglycemia through the cleavage of insulin granules [31]. In this regard, treatments for fasting and high glucose levels resulted in insulin secretion upon Tat-beclin-1-induced autophagy [27]. However, the related mechanisms are unknown and thus need to be studied further. While another study showed that in the presence of high glucose, the rate of autophagic flux in β cells is high, so suppression of autophagy is not applicable [27].

Furthermore, it has been shown that genetic disruption of autophagy results in increased proinsulin content and a simultaneous increase in the secretion of proinsulin and insulin. Conversely, autophagy induction inhibited insulin secretion [27,32]. Thus, it can be concluded that autophagy usually sustains the secretion of proinsulin and insulin. On the other hand, these findings further support that lysosomal degradation has a more critical function in proinsulin and insulin homeostasis. However, Pasquier et al. attributed the degradation of insulin secretory granules to a lysosomal-dependent mechanism (maybe crinophagy) independent of macroautophagy and autophagosome formation [33].

### 3.2. Mitophagy

Mitophagy is an organelle-specific type of autophagy, and maintenance of its integrity is vital for cell survival. Generally, mitochondria are vulnerable to ROS. In addition, these organelles are the center of electron transfer and thus produce abundant oxygen radicals [34]. Therefore, mitophagy is critical for maintaining mitochondria function to sustain energy balance and protect against oxidative stress. Previous studies have shown that selective fusion and segregation of dysfunctional mitochondria and their fission could result in mitophagy and the removal of dysfunctional mitochondria in β cells [35]. Mitophagy can be mediated by two molecular players: PTEN-induced kinase 1 (PINK1) or the E3 ligase Parkin. Parkin, a protein encoded by the *PARK2* gene, is an E3 ubiquitin ligase critical for triggering mitophagy. Parkin-mediated mitophagy, and its upstream regulators, have previously been shown to protect stressed β cells; meanwhile, unregulated mitophagy resulted in the dysfunction of the β cells [36,37,38,39].

It has also been demonstrated that during the diabetes onset in both STZ-induced T1D and *db*/*db* of type 2 diabetes (T2D) mouse models, the p53 protein increases in the β cells, which leads to the inhibition of mitophagy through the PINK1/PERKIN pathway, which results in β cell mass reduction, especially in T2D [40]. Sidarala et al. reported that free radical species-induced inflammatory cytokines mediate mitophagy in β cells through several stages, including the loss of mitochondrial membrane potential (Δψm), translocation of Parkin into mitochondria, turnover of proteins in the outer mitochondrial membrane (OMM), mitochondrial segregation, and, finally, mitochondrial localization to lysosomes for their elimination. Furthermore, they described a protective function for the T1D candidate gene *CLEC16A* in inhibiting β cell death upon inflammatory stress. In other words, they concluded that by controlling mitophagy, *CLEC16A* deficiency made β cells susceptible to inflammation-induced apoptosis and, finally, the development of T1D. Thus, it seems that targeting this pathway can be a therapeutic strategy. In addition, some mitophagy-activating compounds, such as urolithin A, have improved the metabolic function of β cells [41,42,43,44]. Thereby, using pharmacologic inducers of mitophagy may help alleviate inflammatory stress and inhibit β cell dysfunction in diabetes.

### 3.3. ER Stress

**Figure 4:**
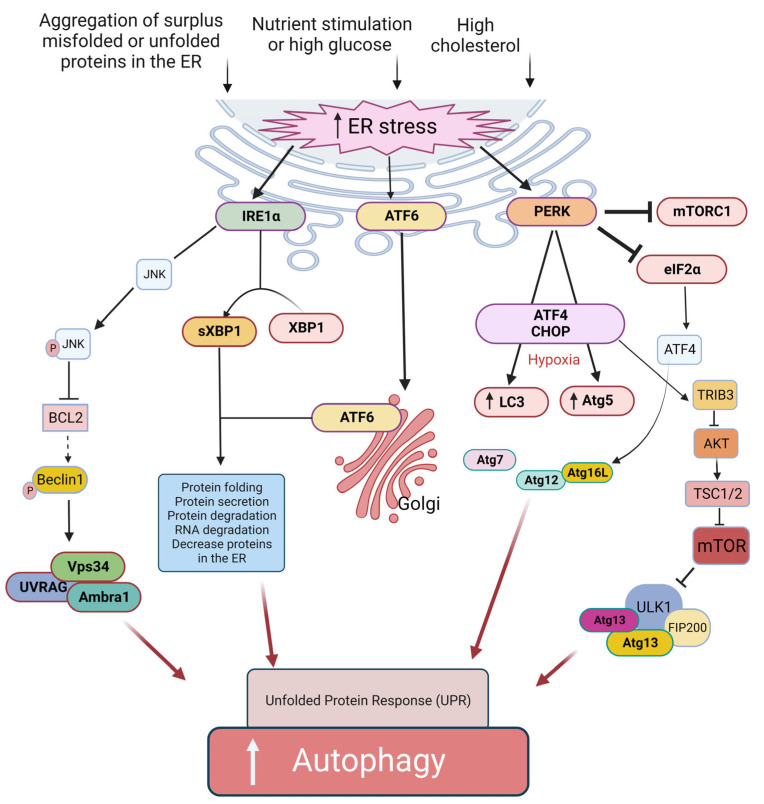
Proposed ER-related mechanisms in the induction of autophagy and UPR in β cells. The ER stress can induce autophagy through PERK, IRE1α, and the ATF6α signaling pathway. The ER stress pathway is caused by hyperglycemia and upon accumulation of misfolded and unfolded proteins in the ER lumen. Activation of IRE1α results in the generation of active sXBP1. The ATF6α is transported to the Golgi apparatus, where it is activated. sXBP1 and the activated ATF6α cause decreased protein burden in the ER. Activation of IRE1α also triggers the JNK signaling cascade, which in turn leads to disruption of the Bcl-2/Beclin-1 interaction through phosphorylation of Bcl-2 and, therefore, induces autophagy. The IRE1α branch of UPR activation of JNK causes phosphorylation of Bcl2, which results in the dissociation of Beclin-1 and, thus, autophagy induction. Another arm of UPR-activated PERK induces autophagy via expression of ATG12 and ATG16L via ATF4 transcription factor; similarly, CHOP activates TRIB3, which blocks the activity of AKT/mTOR pathway-induced autophagy. ATF6α branch of UPR can also induce autophagy by inhibiting phosphorylation at AKT and mTOR pathways.

The ER in β cells has various functions, including producing, processing, and transporting proteins and lipids. During nutrient stimulation and to respond to the increased demand for a sufficient supply of insulin, β cells are forced to significantly enhance their protein synthesis capacity, which imposes a significant burden on the ER. In other words, high glucose level and aggregation of surplus misfolded or unfolded proteins in the ER causes ER stress. The induction of ER stress may present a protective (induction of autophagy) or a cytotoxic (activating apoptosis) mechanism. ER stress can induce the unfolded protein response (UPR) pathway [45]. UPR is an adaptive protective mechanism against ER stress, which prevents cell death by reducing protein misfolding and its outcomes. Three transmembrane sensors in the ER are involved in the regulatory roles of UPR, including protein kinase-like ER kinase (PERK), activating transcription factor 6 (ATF6), and inositol-requiring enzyme 1α (IRE1α) [45] (Figure 4). Indeed, all three pathways of the UPR regulate autophagy. During ER stress, eukaryotic translation initiation factor 2α (eIF2α) is inactivated by PERK. The outcome of this action will be the inhibition of protein translation and a decrease in ER workload [46]. In addition, the activated IRE1α generates an active transcription factor known as splid XBP-1 (sXBP1) by cleaving X-box binding protein 1 (XBP1) transcript [47]. Moreover, IRE1α induces decreased ER workload [48] through insulin mRNAs in β cells [49], thereby reducing the number of proteins in the ER. Simultaneously, the ATF6 protein is activated by translocation into the Golgi [50]. The active forms of sXBP1 and ATF6 (ATF6N) are essential for the upregulation of many target genes that contribute to protein folding, secretion, and ER-related protein degradation [51] to ameliorate ER size and function to reduce ER stress and inhibit cell death [47,50]. Therefore, autophagy activation under ER stress seems to be mediated by the three mentioned signaling pathways: IRE1a/JNK [15], PERK/eIF2a [16], and AKT/mTOR signaling pathway [17]. IRE1α -Xbp1 and PERK-eIF2α pathways can directly induce the expression of autophagic components [52,53,54] (Figure 4). Furthermore, IRE1α can also agitate autophagosome formation by phosphorylating Beclin-2 via JNK activation [55]. In addition, PERK may induce autophagy by inhibiting mTORC1 in the CHOP-Trib3 axis [56]. Additionally, the unphosphorylated PERK (uPERK) is activated under hypoxic conditions, which then upregulates the LC3 and Atg5 through the activating transcription factor 4 (ATF4) and C/EBP homologous protein (CHOP) molecules leading to enhanced autophagy [52] (Figure 4). 

In addition, it has been shown that cholesterol can activate both autophagy and ER stress signaling in β cells, probably via an ER stress-induced PERK/eIF2α signaling pathway [57].

As mentioned, autophagy induction can promote β cell survival under ER stress [9]. It has been shown that Bafilomycin A1 and chloroquine treatment significantly increased β cell death by inhibiting autophagosome formation in primary islet cells affected by ER stress. In agreement with this, it has been illustrated that inhibition of autophagy under short-term stress conditions or acute cytokine induction [58] did not initiate apoptosis, whereas prolonged stress-induced cell death apoptosis. However, it is also reported that the rapid activation of apoptosis may prevent the beneficial effects of stimulated basal autophagy in INS-1 832/13 cells [58]. Moreover, it has been shown that deletion in an autophagy-related protein Atg7, a critical enzyme in the biogenesis of autophagosomes, decreased proliferation and increased apoptosis in mice β cells [8]. This loss of β cell mass was accompanied by reduced insulin production and impaired glucose tolerance [8].

## 4. Modulators of Autophagy in β Cells

### 4.1. Inflammatory Cytokines

Inflammation-related cytokines, including TNF-α, IFN-γ, and IL-1β, are involved in T1D development by increasing reactive oxygen species (ROS) levels and stimulating ER stress. However, their roles are complicated and context-dependent [59,60,61,62]. For instance, it has been demonstrated that IFN-γ and IL-1β induce autophagy in early phases by activating AMPK under ER stress. In addition, these factors also inhibit autophagic flux in β cells by impairing lysosomal function, consequently resulting in apoptosis [63]. Cytokines also increased autophagy preservation in favor of β cell survival. IL-22 showed cell protection against the damage induced by ER stress-mediated palmitate through autophagy induction [64]. Linnemann et al. have recently shown that IL-6 induces autophagy at basal levels and can protect β cells against apoptosis caused by TNF-α, IFN-γ, and IL-1β [65].

### 4.2. Hypoxia

Hypoxia-inducible factor-1α (HIF-1α) is a transcription factor that is upregulated under conditions of hypoxia to provide cellular responses [66]. Indeed, the HIF-1α level is induced by stimulators of hypoxia and infection, including inflammation, cytokines, ROS, and decreased iron levels [67,68,69].

Mitochondria have been shown to regulate autophagy by ROS production [70,71], thus contributing to autophagosome formation and autophagic degradation. In a negative regulatory feedback action, autophagy can also reduce oxidative lesion and ROS levels, for example, by degrading damaged organelles and protein aggregates [72,73]. It has been shown that various molecular players control the ROS-autophagy interaction. For instance, upon excessive production of ROS in mitochondria, the HIF-1α is activated and then induces the transcription of BNIP3/NIX molecules [70]. Furthermore, PERK, as an ER stress sensor, is induced by ROS. PERK, in turn, activates its downstream effectors, resulting in the upregulation of genes involved in autophagy.

HIF-1α-mediated BNIP3 expression is crucial for hypoxia-induced mitophagy [74]. On the other hand, HIF-1α upregulates the BNIP3 gene expression, which binds to the BCL2 competitively with Beclin-1. Upon releasing Beclin-1 from BCL2 and the formation of the BNIP3-BCL2 complex, mitochondrial autophagy is induced to promote cell survival [75]. However, finding the exact relationship between ROS, HIF-1α, and autophagy in the function and fate of the β cell requires further investigations. It has been hypothesized that ROS probably aggravates hypoxia conditions. Subsequently, the activated HIF-1α induces several molecular pathways, such as autophagy [76].

It has previously been reported that overexpression of HIF-1α ameliorates β cell function in a C57BL/6 mouse model [77]. HIF-1α knockdown in β cells increased the susceptibility of NOD mice to T1D exposed to coxsackie viral infection. Furthermore, HIF-1α deletion in β cells was accompanied by impaired viral clearance and increased pancreatitis [78]. In other words, the enhanced viral load and pancreatitis increased the islet and β cell death rate, which can initiate autoimmunity. It has been demonstrated that acute pancreatitis induces HIF-1α [79]. Correspondingly, the induced HIF-1α helps maintain oxygen homeostasis and control genes involved in cell-cycle and apoptosis events [79,80,81,82]. However, in β cells with a deletion in HIF-1α, lack of this protective effect leads to increased β cell death and progression of T1D. Viruses and β cell toxins, including STZ and alloxan, induce cytokines, chemokines, and ER stress, resulting in β cell apoptosis [64,83,84]. Lack of HIF-1α in β cells of mice resulted in progressive immune infiltration, exocrine pancreatitis, and an increased number of cytotoxic T lymphocytes, B cells, and macrophages in islet β cells. Furthermore, genetic association studies indicate an association between polymorphisms in the HIF-1α gene and human diabetes [85,86]. Thus, it seems that HIF-1α plays a critical role in maintaining β cells against autoimmune diseases, such as diabetes, stress, and viral infection. Furthermore, HIF-1α might have a protective role in cells by upregulating the downstream protein BNIP3 and, subsequently, inducing mitochondrial autophagy [87]. Although clear evidence of HIF-1α and autophagy in the β cells has not been documented, HIF-1α might also be involved in β cell autophagy, which will require further studies. Finally, although no available data has yet been reported on the role of HIF-1α, it could be speculated that a ROS-HIF-1α-autophagy cascade plays a role in autophagy (mitophagy)-mediated β cell dysfunction and diabetes pathogenesis. More studies are needed to clarify such crosstalk.

### 4.3. Dietary Constituents

The organelles, including ER, Golgi, mitochondria, lysosomes, and autophagosomes, have substantial regulatory roles in β cell function. In T2D, persistent exposure to excess nutrients and pro-inflammatory cytokines induces dysfunction of these organelles, resulting in β cell dysfunction and disease development [88]. The organelle homeostasis depends on nutrient-sensing molecules such as mTOR and O-linked N-acetylglucosamine transferase (OGT). Their acute activation raises the organelle function in response to the nutrient surplus. However, long-term nutrient excess (i.e., glucose-lipotoxic conditions) and inflammation result in the inability of β cell organelles to perform their functions efficiently and lead to apoptosis. Many researchers have investigated mTORC1 hyperactivity in the β cells to determine its effect on autophagy dysfunction [88,89]. However, more studies are needed to clarify the role of these nutrient sensors in autophagy dysfunction in the β cell during T2D, which will help manage and find efficient treatments for diabetes in the context of autophagy dysfunction.

#### 4.3.1. Surplus or Restricted Access to Nutrients

It has been demonstrated that surplus and restricted β cells’ nutrient access can induce autophagy. For example, calorie intake changes in mice, from a high-fat diet to a nutrient-restricted diet, led to an increased p62 degradation and LC3II/LC3I ratio as two primary autophagy-related markers [90]. Moreover, a later study reported that nuclear factor erythroid 2-related factor 2 (NRF2), an antioxidant factor, is crucial for β cells’ response to temporary high-fat intake changes [91]. The transcription factor NRF2 regulates the expression of detoxifying, including GSTμ and GSTθ, and antioxidant enzymes [92]. Under normal conditions, this protein is degraded through the ubiquitin-proteasome system via the action of an adaptor of the ubiquitin ligase complex known as kelch-like ECH-associated protein 1 (KEAP1). The KEAP1-NRF2 pathway is one of the principal cellular defense machines responding to oxidative stress [92,93]. In mice lacking β cell autophagy, oxidative damage increases, resulting in the loss of such antioxidant-protective pathways (because of the failed compensatory increase in NRF2), increased β cell apoptosis, and, subsequently, diabetes onset. This leads to intranuclear translocation of NRF2 to induce the expression of many antioxidant genes [92,93]. NRF2 contributes to the autophagic process in response to oxidative stress and functions in a feedback loop in combination with AMP-activated protein kinase (AMPK), which is critical for autophagy induction through mTOR down-regulation [94]. It has been shown that a high-fat diet (HFD)/oxidative stress induces the NRF2/antioxidant pathway. Thus, because of the NRF2 role in the induction of autophagy, it may be concluded that NRF2 is a crucial factor for β cells’ survival against stress conditions, especially in severe alterations in nutrient intake.

Starvation and nutrient deprivation are known autophagy inducers; however, discrepancies exist regarding their effect on β cells. Long-lasting starvation resulted in autophagy induction compared with studies where the duration of nutrient deprivation was restricted. Goginashvili et al. [31] reported that short-term starvation might suppress autophagy and induce crinophagy in β cells. Based on these findings, it may be concluded that restricted starvation may activate crinophagy, while a long-lasting nutrient-restricted strategy results in macroautophagy stimulation in the β cell. Although several in vitro and in vivo studies have reported β cells autophagy induction following starvation and fasting [7,95,96,97], others did not observe such an effect [7,31]. On the other hand, Goginashvili et al. reported that starvation of pancreatic cells resulted in macroautophagy suppression compared to many other mammalian cell types. Alternatively, starved cells caused lysosomal degradation of nascent secretory insulin granules, a mechanism regulated by protein kinase D (PKD), an essential regulator of secretory granule biogenesis. They concluded that this phenomenon caused lysosomal recruitment and mTOR activation, which suppressed macroautophagy, a crucial switch for keeping insulin secretion low during fasting. Ebato et al. [7] also discovered that starved C57BL/6 mice showed no increase in autophagic vacuole formation in β cells of islets isolated from C57BL/6 mice close to those seen in C57BL/6 mice fed a regular chow diet. They further concluded that β cell autophagy is significantly increased by high-calorie intake than energy starvation, a joint state observed in many developed countries. These contradictory results seem to be ascribed to the duration and time of starvation and the type of activated autophagy.

#### 4.3.2. Fatty Acids, Supplements, and Hormones

In vitro studies have shown that autophagy protects β cells against apoptosis stimulated by fatty acids, including cholesterol and palmitate [98,99,100]. Meanwhile, a high glucose and lipids diet can cause β cell death by suppressing autophagy due to lysosomal defects [101,102,103]. Specifically, animal studies have shown that excess fat can stimulate autophagy, while a combination of high fat and glucose inhibits autophagy in β cells [91,104,105]. It has been demonstrated that mitogen-activated protein kinases (MAPKs) contribute to the pathogenesis of obesity-induced insulin resistance and T2D [104]. The MAPK phosphatase (MKP) family acts as the upstream regulator of MAPKs. The MKP-5 is considered a new regulator of extracellular signaling that controls lipid metabolism. Zhao et al. [106] showed that MKP-5 protects islet cells from HFD-fed mice against lipotoxicity-induced apoptosis and dysfunction by increasing autophagy flux mainly via the negative regulation of the P38 and JNK/MAPK signal transductions. By treating the Rin-m5f cells with the autophagy inhibitor 3-methyladenine (3-MA), they also demonstrated that overexpression of the MKP-5 suppressed the palmitic acid (PA)-induced apoptosis, inflammatory response, and oxidative stress through ameliorating autophagy [106].

Therefore, it may be concluded that prolonged exposure to high fatty acids and glucose can impair the β cells’ protective mechanisms (here autophagy) against stressful conditions, which, consequently, results in diabetes progression.

Recent investigations have shown that dietary constituents such as secondary metabolites and vitamins can promote autophagy in β cells to protect them against stress. For example, kaempferol, a natural flavonoid, exhibited a protective effect for β cells by activating autophagy via the AMPK/mTOR pathway in a T2D model with high palmitic acid administered [107]. Another natural flavonoid, quercetin, reduced palmitate-induced β cell apoptosis by restoring lysosomal function and autophagic flux [108]. In addition, it has been shown that omega-3 and vitamin D administration decreases the incidence of T1D in mice treated with streptozotocin (STZ) and protects β cells against STZ-induced apoptosis through upregulation of Atg7 [109,110,111]. These findings suggest that the natural nutrient supplement and antioxidants have autophagy regulation potential and can be considered for autophagy induction in β cells in therapeutic applications.

Glucagon-like peptide-1 (GLP-1) hormone induced glucose-mediated insulin secretion and was shown to promote β cell proliferation and autophagy [110]. In addition, the agonists of the GLP-1 receptor, exendin-4 and liraglutide, resulted in induced autophagy and protection in both INS-1 cells and pancreatic islets against lipo- and glucolipotoxicity [112,113]. Furthermore, administration of the GLP-1 receptor agonists or inhibition of the GLP-1 inhibitor (DPP-4) in mice fed HFDs and rats treated with tacrolimus showed protective effects in β cells by stimulating autophagy [113,114].

## 5. Type 1 Diabetes

The role of autophagy in the pathogenesis of T1D needs to be better understood, and only a few studies are available [115,116]. T1D is characterized by β cell destruction, probably due to pro-inflammatory cytokines and autoreactive T cell invasion in the pancreatic islets during the early stages [117]. It is hypothesized that the proteolytic pathways, including autophagy, contribute to the etiology of T1D. In the early stages, environmental factors can cause the breakdown of self-proteins to target β cells. Autoantigens are trimmed to modified peptides in β cells and loaded into MHC class I molecules. The peptide-MHC class I complex (pMHCI) reaches the progenitor membrane and is recognized by the T cell receptor (TCR) on CD8 + T cells, causing T cell activation and β cell lysis. Antigen-presenting cells (APCs), such as dendritic cells, are recruited within the gland to take up altered autoantigenic fragments transported to the influx area lymph nodes. APC encounters T and B lymphocytes, resulting in the attack of initial β cells and the production of autoantibodies that continue to circulate throughout the preclinical stage of the disease. At the clinical onset, autoreactive T and B lymphocytes are further expanded and activated under the action of a second trigger event, e.g., viral injury or other environmental factors, resulting in local inflammation known as “insulitis” and massive β cell destruction. This event could drive autoimmunity against the pancreatic β cells and their destruction. Indeed, several studies have indicated the possible role of autophagy in both MHC class I and MHC class II peptide presentation in T1D pathogenesis [118]. Perturbation of the thymic selection of T cells during the initiation phase of the disease and autophagy’s role in this process has been suggested as the potential cause [119]. However, it is unclear if defects in the immune cells or autophagy defects in the β cells have a significant role in the events leading to T1D.

**Figure 5:**
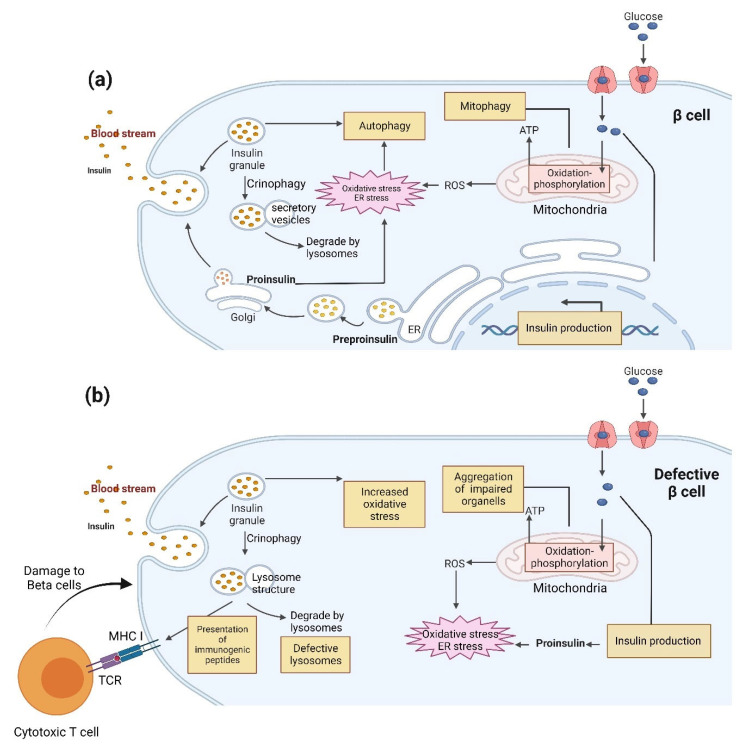
The possible role of autophagy (crinophagy) in insulin production, physiological conditions, and the pathological onset of T1D. (**a**) Glucose metabolism in the mitochondria generates ATP and ROS, which are two important insulin production and secretion stimulators. Hyperglycemia can lead to excessive ROS levels, leading to oxidative stress and the increased burden of chronically high levels of insulin secretion on the ER, resulting in ER stress and oxidative stress, which autophagy can alleviate to protect the β cells against apoptosis. As an alternative, it may be possible that stimulated crinophagy leads to direct insulin granules fusion to lysosomes instead of autophagy. In the physiological condition, protein degradation generated peptides tailored in the ideal length for binding to MHC-I and recognized as self and tolerated. This occurs through the ubiquitin/proteasome system or alternative processes such as autophagy. (**b**) In the early stages of T1D pathogenesis, environmental factors can cause the breakdown of self-proteins to target β cells. Autoantigens are trimmed to modified peptides in β cells and loaded into MHC-I molecules. The pMHCI reaches the progenitor membrane and is recognized by the TCR on CD8 + T cells, causing T cell activation and eventually insulitis.

Autophagy is vital for MHC-I and II self-antigen presentations [119]. Increased MHC-I expression during impaired β cell autophagy, heterogeneous presence of MHC-II on β cells of T1D organ donors, and formation of highly antigenic chimeric epitopes by transpeptidation in β cell secretory granule proteins via the activity of lysosomal cathepsin proteases are reported among probable pathogenic causes [120,121,122]. Some evidence suggests direct communication of β cells with diabetogenic CD4+ T cells [120]. Generally, in β cells, peptides are generated in the ideal size for binding to MHC-I via autophagy’s ubiquitin/proteasome system [123]. Loss of autophagy might cause inhibition of MHC presentation of pancreatic autoantigens, including insulin, glutamic acid decarboxylase (GAD), and insulinoma-associated antigen 2 (IA2), to medullary thymic epithelial cells [123]. It is hypothesized that defective autophagy can alter the load on MHC-I molecules in β cells (Figure 5).

Dysfunctional lysosomes might impair autophagy in T1D, potentially affecting several autoimmunity-related mechanisms. For instance, it has been presumed that releasing incompletely digested peptides, such as insulin, could stimulate autoimmune invasion [124]. Many lysosomal hydrolases are carried to the lysosomes via the Mannose-6-Phosphate Receptor (M6PR) in a cation-independent manner, and deficiency of M6PR is correlated with increased susceptibility to β cell stress [125]. Studies have shown that deletion of *Atp6ap2*, a subunit of the vacuolar ATPase required for lysosomal degradation and autophagy, disturbs β cell lysosomal acidity, resulting in inadequate insulin secretion, impaired autophagic flux [126,127], and, finally, β cell dysfunction. Another aspect pointing towards lysosomal dysfunction in T1D is our observation that several of the lysosomal cathepsin proteases are differentially expressed in human islets and β cell lines in response to pro-inflammatory cytokines, suggesting that immunomodulation of cathepsin expression may contribute to immune-mediated β cell dysfunction in T1D [128]. Recent studies also indicate that dysfunctional autophagy caused by impaired lysosomal function and leakage of lysosomal cathepsins contributes to cytokine-induced β cell apoptosis [63,126,129]. Notably, Muralidharan et al. recently reported impaired autophagy in residual β cells of both NOD mice and human donors with T1D [129]. Some of the lysosomal cathepsin proteases are genetically associated with T1D, i.e., cathepsin B (CTSB) and H (CTSH) [130,131,132], and some are modulators of β cell survival. We previously reported that cathepsin C (CTSC) and CTSH have anti-apoptotic functions in β cells [128,133,134], whereas CTSB is pro-apoptotic [63].

Thus, it has been suggested that targeting the lysosome by selective induction of autophagy and/or lysosome function in β cells may be a feasible strategy to restore homeostasis and function for T1D prevention, especially in the early prediabetic stage. Pasquier et al. also suggested that the macroautophagy-independent lysosomal degradation of nascent insulin granules is remarkably increased under metabolic stress. In other words, stress-induced nascent granule degradation (SINGD) results in insulin loss and mTOR-dependent macroautophagy dysfunction in diabetic β cells [33].

In individuals with T1D, evidence of reduced crinophagy and macroautophagy impairment was observed in the β cells. Indeed, it has been suggested that crinophagic bodies contain short peptides with immunogenic epitopes. Thus, the altered crinophagy could result in the exposure of modified peptides that recruit T cells to the β cells [135]. In agreement with this, detectable levels of proinsulin in the blood of T1D patients [136] and elevated proinsulin levels in the β cells with a deficiency in autophagy [27] have been reported. Furthermore, recent studies on diabetic in vivo models, such as NOD mice, have also shown that the defective clearance of autophagosomes in β cells could impair β cell autophagy in T1D [129]. This study showed reduced autophagosomes fusing with lysosomes and impaired autophagic flux in diabetic NOD mouse islets. These findings may suggest a possible function of impaired crinophagy and autophagy in T1D. Thus, it is plausible to accentuate that selective induction of autophagy and/or lysosome function may be beneficial in restoring β cell function during the initial prediabetic period.

## 6. Type 2 Diabetes

T2D results from insulin resistance and β cell dysfunction. In response to insulin resistance, the insulin secretion capacity of pancreatic β cells is enhanced (known as hyperinsulinemia) to relieve the effect of hyperglycemia during the early stage of T2D. However, because of the metabolic pressure and increasing insulin demand, β cell mass decreases due to apoptosis during T2D progression [137,138,139,140]. Both increased and decreased levels of autophagy have been reported in metabolically stressed β cells in diabetes. Autophagy is increased in β cells in pre-diabetes, which can be protective, while autophagy decreases as diabetes progresses, resulting in β cell failure [7,102]. Deficiency in autophagy and related pathways in β cells has recently been shown to be causative in the progression of T2D. Indeed, autophagy contributes to reducing β cell mass in T2D [139]. The lysosome-associated membrane proteins (LAMP) and cathepsins are involved in several steps of autophagy, especially the autophagic flux. Masini et al. presented evidence of changed autophagy in β cells obtained from samples of individuals with T2D, including accumulation of autophagic vacuoles and autophagosomes and reduced LAMP2 and CTSB and cathepsin D (CTSD) gene expression [141]. In support of this, Cnop et al. reported differential expression of several cathepsins in human pancreatic islets after exposure to palmitate [142], suggesting that dysregulated cathepsin expression contributes to β cell dysfunction in T2D.

Several studies have shown that activated autophagy is protective during oxidative stress in β cells in T2D [143,144]. Indeed, β cells in Atg7 knockout mice with T2D showed deficient basal autophagy, reduced β cell mass, decreased insulin secretion, upregulated p62, and apoptosis [2]. Moreover, in vivo models of Atg7-deficient mice [145], the β cell-specific Atg7 knockout mice, and the *db*/*db* mouse model have revealed the vital role of autophagy in the progression of diabetes as well as in preserving the function of pancreatic β cells. The evidence includes a reduced number of β cells, glucose intolerance, reduced level of insulin secretion, and accumulation of autophagosomes in the pancreatic β cells in these T2D models [7,145,146,147,148,149]. Furthermore, detrimental effects on β cells resulting in autophagic cell death due to chronically activated autophagy during T2D progression were also reported [7,150,151,152,153].

Deficient autophagy can also result in β cell damage by the accumulation of human islet amyloid polypeptide (hIAPP), which plays an essential role in T2D pathogenesis. In other words, autophagy is indispensable for protecting the β cells by clearing the hIAPPs [154]. Deficient autophagy could also lead to the accumulation of impaired organelles, including mitochondria [155]. In T2D, a large surplus of autophagic vacuoles and autophagosomes could lead to the loss of β cell mass [156].

It has been suggested that forkhead box transcription factor O1 (FoxO1)-mediated autophagy increases cell viability and suppresses cell apoptosis in various cells [157,158]. Previous studies indicate that FoxO1 is also important for pancreatic β cell survival and insulin secretion, e.g., via autophagy (reviewed in [159,160]). In a recent study, FoxO1 silencing in INS-1 cells led to impaired autophagy and reduced cell viability [161]. Some hypoglycemic drugs, including GLP-1 receptor agonists and GLP-1 analogs, have been reported to stimulate β cell autophagy, e.g., via FoxO1, thereby protecting against β cell stress [162,163]. Therefore, regulation of the FoxO1-autophagy axis in islet β cells could be a novel therapeutic strategy in T2D.

## 7. Autophagy-Related Genes Genetically Associated with T1D and T2D

Genetic variations in autophagy-related genes have been shown to affect the autophagy process in human cells, leading to multiple pathological changes and eventually causing susceptibility to various human diseases [164,165,166]. A quick survey of all known human autophagy genes revealed associations with several T1D and T2D loci (Table 1). Ten and eighteen autophagy-related genes were genetically associated with T1D and T2D, respectively, with *INS* as the only common candidate gene (Table 1). All 27 genes are expressed in human β cells and, with the exception of SEC16B, in whole human islets (Table 1). Nine genes showed differential expression in response to the β cell stressors free fatty acids and/or pro-inflammatory cytokines (Table 1). One is *CTSB*, which has been suggested as a molecular linker between autophagy and the NLR family pyrin domain containing 3 (NLRP3) inflammasome in β cells [167,168], which is important for the inflammatory response as well as cell death.

Previous studies have shown an association of autophagy-related genes with diabetes, e.g., *CLEC16A* and *PTPN2*, which were associated with T1D in previous GWAS studies [169,170] but not in the most recent GWAS meta-analysis [132]. *CLEC16A* regulates the NRDP1/PARKIN pathway, which is a master regulator of mitophagy. Loss of *CLEC16A* in mice resulted in decreased insulin secretion and increased ER stress, thereby decreasing the overall β cell function, thus supporting a protective role for *CLEC16A* in β cells via its regulation of mitophagy [171]. The *CLEC16A* intronic T1D SNP rs12708716 was associated with reduced islet *CLEC16A* expression and impaired glucose control in 80 healthy human islet donors [171]. Another T1D SNP, rs1893217, within the intronic region of *PTPN2* has been linked to impaired autophagosome formation and defective bacterial handling in macrophages and intestinal epithelial cells [172]. Moreover, the T1D candidate gene *CTSH* encodes a lysosomal cysteine cathepsin involved in the degradation stage of autophagy. We have previously shown that T1D SNPs within *CTSH* regulate the expression of CTSH and are associated with disease progression in children with newly-diagnosed T1D [133]. 

A recent genome-wide CRISPR screening study in EndoC-βH1 cells by Rottner et al. demonstrated that the T2D candidate gene *CALCOCO2*, encoding a cargo receptor that recruits the degradation target to the autophagic machinery, regulates insulin granule homeostasis [173]. Silencing *CALCOCO2* in EndoC-βH1 cells resulted in reduced insulin content via an autophagy-based reduction in proinsulin and immature granules, and T2D SNPs associated with *CALCOCO2* affected insulin secretion in human islets [173].

Further studies are warranted to identify novel associations for genetic variants within autophagy-related genes that might shed light on the roles of autophagy in regulating β cell function and metabolic traits.

**Table 1:** Autophagy-related genes genetically associated with T1D and T2D. Human autophagy-related genes were extracted from the Human Autophagy Database on 18 March 2023 (http://autophagy.lu/) and the Autophagy Database on 18 March 2023 (http://www.tanpaku.org/autophagy/) and checked for the genetic association to T1D and/or T2D based on the most recent GWAS meta-analyses [132,174]. Expression levels are shown as counts per million (CPM) in human islets and FACS-sorted human β cells based on data from previously published studies [175,176,177]. A CPM > 1 was used as the cut-off level for expression. Regulation by free fatty acids (FFA) and/or pro-inflammatory cytokines (CYT) was based on previously published studies in human islets [142,178].

Gene	Genetic Association	Expression in Human Islets (Average CPM)	Expression in Human β Cells (Average CPM)	Regulated by β Cell Stressors in Human Islets (FFA/CYT)	Cellular Localization
*ANK1*	T2D	7.35	26.55		Membrane
*CAMK1D*	T2D	21.68	19.86		Cytoplasm
*CDKN1B*	T2D	30.71	63.32		Nucleus
*CDKN2A*	T2D	7.64	13.58	FFA	Nucleus
*CELF1*	T2D	181.06	176.65	CYT	Cytoplasm
*CTSB*	T1D	244.49	343.90	CYT	Cytoplasm
*INS*	T1D, T2D	6889.44	9219.81		Extracellular Space
*INSR*	T2D	124.92	53.21		Membrane
*IRS1*	T2D	52.77	21.37		Cytoplasm
*IRS2*	T2D	117.07	83.20	FFA	Cytoplasm
*ITPR2*	T2D	28.51	59.25		Cytoplasm
*MAP2K5*	T2D	10.71	12.43		Cytoplasm
*MAP2K7*	T2D	50.40	20.02		Cytoplasm
*MAP3K14*	T1D	48.85	25.07		Cytoplasm
*MAPK3*	T1D	48.00	21.80		Cytoplasm
*MARK3*	T2D	89.10	83.86	CYT	Cytoplasm
*MTMR9*	T1D	23.24	66.30		Cytoplasm
*MYC*	T1D	79.04	4.50	FFA, CYT	Nucleus
*NFKB1*	T1D	180.36	40.06	FFA, CYT	Nucleus
*PTEN*	T2D	80.47	281.77		Cytoplasm
*SEC16B*	T2D	-	1.54		Nucleus
*SLC1A2*	T1D	9.65	12.91		Membrane
*SLC7A7*	T2D	7.38	9.28	CYT	Membrane
*SPG7*	T2D	66.67	83.12		Cytoplasm
*TBC1D4*	T1D	39.84	142.60	CYT	Cytoplasm
*TRAF1*	T1D	80.53	13.98		Cytoplasm
*VEGFA*	T2D	316.32	537.68		Extracellular Space

## 8. Autophagy as a Therapeutic Target

Autophagy’s critical role in β cell differentiation, reprogramming, and function makes it a potential therapeutic target [13,179,180]. Thus, targeting the molecular players and related pathways in autophagy might be an efficient strategy for diabetes therapy.

Dietary modulation, including caloric restriction, is one of the promising strategies as an autophagy intervention that can be useful in preventing and treating diabetes. Studies on mice have shown that a fasting-based diet could enhance the β cell regeneration in T1D and T2D [180]. Furthermore, the “fasting” strategy in the islets isolated from patients with T1D restored insulin production [181]. It has also been shown that the administration of complementary dietary ingredients, including vitamin D, and the standard insulin treatment in T1D patients might be helpful for the function of the residual β cells [140].

Studies have also shown that metformin, GLP-1 analogs, and rosiglitazone, as effective therapeutic agents for diabetes, induce autophagy in the β cells. Metformin has been used to induce autophagy and inhibit apoptosis in β cells under lipotoxicity [182]. Rosiglitazone has also been shown to stimulate β cell autophagy and protect them against palmitate-mediated apoptosis [183]. Furthermore, it has been demonstrated that GLP-1 could protect β cells via induction of autophagy. Thus, GLP-1 analogs, such as GLP-1 receptor agonists and DPP4 inhibitors, are being investigated to elucidate their potential in diabetes treatment, especially T1D [184].

Autophagy suppression using mTORC1 and protein kinase A (PKA) inhibitors also showed similar results. Rapamycin is an immunosuppressant and anti-cancer agent in organ transplantation and cancer therapy. Rapamycin is also a mTORC1 inhibitor that can induce β cell autophagy in vitro and in vivo [185,186,187]; contradictory results have been reported for its antidiabetic potential in rodent models. Several studies have shown that rapamycin can cause weight reduction, decrease blood sugar levels, and decrease insulin resistance in diabetic models [188,189,190,191,192]. Other studies have also reported that it can reduce β cell function and viability [191], decrease glucose tolerance, increase insulin resistance, and stimulate autophagy [188,191,193,194,195,196,197]. Thus, more studies are required to clarify the efficacy of rapamycin in autophagy-related approaches in diabetes therapy.

Depending on the context and period of autophagy inhibition, β cell function is distinctly affected. For instance, short-term inhibition of autophagy in β cells increases ether lipids and peroxisomal function and counters depletion of n-3 polyunsaturated fatty acids upon HFD feeding. Indeed, in response to high-fat feeding, β cells decrease n6:n3 PUFA ratios in ether lipids and phospholipids through short-term inhibition of autophagy. In other words, an intervention study by long-term autophagy inhibition showed impaired glucose tolerance, diminished insulin content, and growth of islet-cell cysts in β cells [7,8]. In contrast, short-term inhibition of autophagy resulted in improved glucose-stimulated insulin secretion.

## 9. Concluding Remarks

Emerging outcomes from cellular experiments, animal models, and human studies suggest the important role of autophagy in β cells’ health. The summarized knowledge in this review indicates the unique association of ‘compromised autophagy’ with β cells’ functional failure and pathologic condition augmentation. Elucidating the autophagy mechanisms and identifying the related genes have played major roles in our understanding of both autophagy’s physiological and pathological role in β cell homeostasis.

Generally, autophagy can be selective and nonselective [198]. For instance, starvation mainly triggers nonselective autophagy, in which the components in the cytoplasm will be degraded. On the other hand, mitophagy, ER-phagy, and lipophagy are examples of selective autophagy. ER-phagy [199] and lipophagy [32] in β cells have not been demonstrated; somewhat due to the lack of sensitive and specific methods to observe these types of autophagy. These processes maintain cellular homeostasis in the β cells under stress conditions [200], insulin biosynthesis pressure, or continuous flow via the ER-Golgi route, which are standard features of endocrine cells, including β cells. Moreover, as discussed, the activation of the autophagic process by ER stress is mediated by elevated Atg12 expression, JNK activation, and mTOR inhibition, revealing the intimate link between ER and autophagy [58,201,202]. The relevance of these observations is unclear; however, it might be linked to an increased requirement for autophagic clearance of misfolded proteins in the ER lumen under ER stress or unfolded protein buildup. Furthermore, it is believed that besides the UPR, autophagy triggered by ER stress might be an alternative degradation process of these misfolded proteins, including misfolded proinsulin dependent or independent of ATF6 and IRE1α [198].

More studies are needed to understand the exact role of autophagy in the β cells and its contribution to diabetes pathogenesis. Additionally, future efforts to discover dietary ingredients and regimens with the potential to induce autophagy and maintain β cells would help facilitate the discovery of proper therapeutic targets for diabetes therapy strategies.

## Data Availability

Not applicable.
